# Impact of daily fasting duration on body composition and cardiometabolic risk factors during a time-restricted eating protocol: a randomized controlled trial

**DOI:** 10.1186/s12967-024-05849-6

**Published:** 2024-11-29

**Authors:** A. Sampieri, A. Paoli, G. Spinello, E. Santinello, T. Moro

**Affiliations:** 1 Department of Biomedical Sciences, University of Padua, Padua, Italy; 2 Department of Medicine, University of Padua, Padua, Italy

**Keywords:** Time-restricted eating, Intermittent fasting, Body composition, Fat mass, Body mass loss

## Abstract

**Background:**

Time-restricted eating (TRE) is a dietary regimen that limits food intake for at least 12 h daily. Unlike other fasting protocols, TRE does not dictate what or how much to eat but rather focuses on the timing of meals. This approach has been previously demonstrated to improve body composition in individuals with obesity or metabolic impairments. However, its impact on body composition and cardiometabolic factors in healthy individuals remains unclear. Furthermore, the optimal fasting duration is still debated. Thus, we aimed to compare the effects of 8 weeks of different fasting durations on body composition and biochemical parameters in metabolically healthy, non-trained individuals using a parallel randomized controlled trial.

**Methods:**

Forty-one volunteers were randomly assigned to one of the four experimental groups: TRE 16:8 (16 h of fasting,8 h of eating), TRE 14:10 (14 h of fasting,10 h of eating), TRE 12:12 (12 h of fasting,12 h of eating) or a normal diet group (ND; no dietary restriction). Participants underwent body composition measurements and blood tests for lipid profiles (i.e., total cholesterol, LDL, HDL, and triglycerides), fasting glucose, leptin, and anabolic hormones (i.e., insulin and testosterone) levels. Data were analyzed using both intention-to-treat (ITT) and per-protocol (PP) analysis to account for compliance. A two-way ANOVA for repeated measures was employed to assess interactions between time and group.

**Results:**

In the ITT analysis, TRE 16:8 reduced body mass (-2.46%, *p* = 0.003) and absolute fat mass (-8.65%, *p* = 0.001) with no changes in lean soft tissue and in calorie intake. These results were consistent with the PP analysis which included 8 participants in TRE 16:8, 5 in TRE 14:10, 9 in TRE 12:12, and the entire ND group. Participants in the TRE 16:8 group spontaneously reduced their total caloric intake, although this reduction was not statistically significant. None of the other measurements significantly changed after 8 weeks.

**Conclusions:**

Our results suggest that a 16-hour fasting window, even without caloric restriction, may be a viable strategy for improving body composition in healthy and non-trained individuals, whereas a shorter fasting period may be insufficient to produce significant changes in a healthy population.

**Trial registration:**

NCT, NCT04503005. Registered 4 August 2020, https://clinicaltrials.gov/study/NCT04503005.

**Supplementary Information:**

The online version contains supplementary material available at 10.1186/s12967-024-05849-6.

## Introduction

According to World Health Organization (WHO) statistics, global adult obesity is steadily rising, with 43% of adults being overweight and 16% living with obesity in 2022 [1]. Overweight and obesity are conditions characterized by excessive fat deposits that impair health. Indeed, they have been associated with the development of chronic diseases such as type 2 diabetes, cardiovascular disease, and certain types of cancer [2–5]. According to the American Heart Association guidelines, a 5–10% reduction in body mass can significantly improve clinical outcomes including lower fasting glycemia, triglycerides, and blood pressure [6]. Therefore, maintaining fat and body mass within a normal range is crucial for health.

Lifestyle modifications are well-recognized as optimal approaches to effective body fat management. Among these, the Mediterranean diet has been proposed as a dietary model for preventing cardiovascular risk and managing body mass [7, 8]. An expanded perspective on the cultural and social dimensions of the Mediterranean diet and other health-influencing forms the basis of the new concept known as the “Planeterranean diet” [9]. Main aspects of these kinds of diets are the consumption of local/seasonal foods and the spontaneous caloric restriction rather than some specific foods [10]. With respect to cultural and social dimensions, it is undeniable that today’s society is characterized by sedentary behavior. Consequently, it is understandable that continuous calorie restriction (CCR), with or without exercise, represents the most common treatment for managing obesity [6]. However, adherence to CCR decreases within the first month and continues to decline in the following months [11, 12] with many individuals regaining a significant amount of weight by the end of one year [5]. Additionally, when CCR is adopted without exercise, there is a significant risk to lose lean soft tissue (namely, skeletal muscle mass) concurrently with fat mass [13, 14]. This loss of lean soft tissue (LST) is particularly concerning because it can lead to decreased total energy expenditure [15], contributing to more challenging maintenance of the new acquired body mass [16]. Another characteristic of the original Mediterranean Diet is the meal frequency: as suggested by some authors, the short fasting periods typical of the 7-day Adventists behaviors, may have had a positive role on many health outcomes [17]. It is noteworthy that physiological fasting periods were embedded in our ancestors’ lifestyles.

Considering its role in human health and its evolutionary implications, alongside the possibility to overcome CCR possibly negative effects on muscle mass, intermittent fasting (IF) has gained considerable popularity over the past decade [18–20]. According to the recent International consensus on fasting terminology, IF is a dietary approach characterized by repetitive fasting periods lasting up to 48 h each, interspersed with a period of regular eating on a cyclical basis [21]. IF has emerged as a promising dietary approach, not only for body mass management or metabolism improvements but also for enhancing compliance, especially for individuals who struggle with conventional calorie-restricted diets [22, 23]. Indeed, unlike traditional CCR diets, which often require meticulous monitoring of food intake, IF provides greater flexibility by focusing primarily on the timing of meals rather than specific food restrictions without affecting diet quality [23].

One specific type of IF is time-restricted eating (TRE), a regimen that allows participants to consume food ad libitum within a defined eating time window each day [24]. According to the above-mentioned recent international consensus, TRE should ideally include a fasting period of at least 14 h; however, some experts propose a more flexible approach, suggesting a minimum fasting window of 12 h [21]. Thus, depending on the length of the eating and fasting windows, TRE protocols can vary and the more utilized formats are 16:8, 14:10, and 18:6 (fasting: eating ratio) [25]. This type of dietary regimen has been shown to produce higher compliance and adherence because calorie intake is not controlled [26, 27]. In contrast to a typical CCR protocol, where daily energy intake is consistently reduced by 20–40% while the meal frequency remains unchanged [28], TRE can be practiced with or without calorie restriction. The key aspect of TRE is that repeated bouts of prolonged fasting (> 12 h) stimulate fatty acid release from adipocytes to overcome hepatic glycogen depletion [29].

The effects of TRE on body composition are well known, and several systematic reviews have documented a decrease in body mass and body fat mass across individuals with different baseline characteristics (e.g., metabolic impairments, obesity) and under different experimental designs (e.g., hours of fasting, with or without exercise) [25, 30–32]. A recent systematic review and meta-analysis showed that LST appears unaffected by TRE regimens [25], while the effects on health markers, including fasting glucose and plasma lipids, remain controversial [25, 30–32].

Despite the general higher compliance of TRE regimes, limiting food intake during a limited time window poses a challenge, especially for a modern lifestyle. Indeed, in industrialized countries, most people eat over a window of more than 15 h, with the meals consumed after 6:30 p.m. contributing substantially to exceeding daily caloric requirements [33]. Thus, reducing the eating window to 8 h (to reach 16 h of fasting) might be drastic, whereas a 10-hour or 12-hour eating window may be more feasible. Hence, in this research, we compared the effects of 16-hour, 14-hour, and 12-hour fasting time-restricted eating regimens versus a no-time-restricted eating control group on body composition and biochemical parameters in healthy non-trained individuals. We hypothesized that the advantages of fasting are more significant with a 16-hour fasting period and, to a lesser extent, with a 14-hour fasting.

Moreover, while most studies have focused on overweight and obese populations, there is limited research on the effects of TRE in normal-weight, metabolically healthy individuals [20]. Therefore, given the widespread adoption of TRE protocols among healthy and normal-weight individuals, largely fuelled by social media attention to this nutritional approach, we deemed it crucial to understand the effects of TRE in this population.

## Materials and methods

### Participants

Forty-eight people living in Padua (Italy) or neighboring areas were reached through social networks and word-of-mouth. Inclusion criteria were: (i) adults aged between 18 and 65 years, and (ii) 18 kg/m^2^ < BMI < 30 kg/m^2^. Exclusion criteria included: (i) engaging in structured physical exercise more than once per week, (ii) diagnosis of metabolic or cardiovascular diseases, (iii) previous experience of any type of TRE, and (iv) change in body mass greater than 5% within the 3 months prior to the study. Based on the previous criteria, three participants were excluded due to type II diabetes and one due to BMI > 30 kg/m^2^, whereas other three participants declined participation after explaining the study’s protocol. Therefore, 41 people were included in the study and randomly assigned to either TRE groups or a standard diet. Randomization was performed by a stratified random sampling procedure by sex, age (18–35 and 36–65 years), and BMI (18-24.9 and 25–29.9 kg/m^2^). The randomization was generated using the online software GraphPad (https://www.graphpad.com/quickcalcs/randomize1/), and a randomization list was created before the start of the study within each stratum. Age stratification is based on the common categorization of young adults and middle-aged individuals due to the well-known effect of aging on body composition, metabolism, and energy expenditure [34, 35]. Thus, the randomization procedure ensured a relatively even distribution of younger and older adults and of normal-weight and overweight individuals. The participants received information about the group allocation after the first visit.

**Fig. 1:**
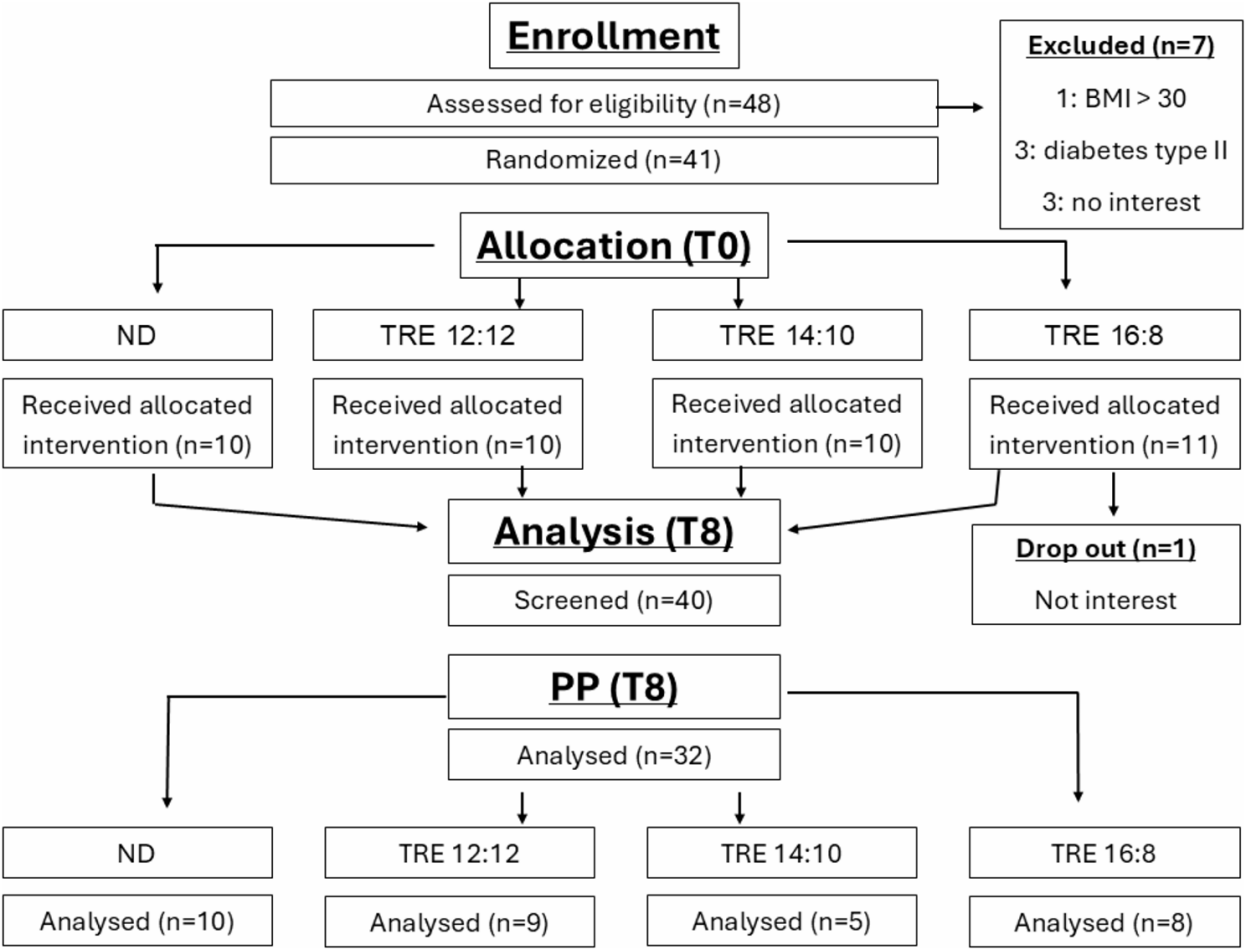
Study flowchart. PP: per protocol

The visits, number of participants, and drop-out are displayed in Fig. 1. The participant’s baseline characteristics are shown in Table 1.

Participants were first explained the protocol and the potential risks. They read and signed an informed consent document approved by the ethical committee of the Department of Biomedical Sciences, University of Padova (HEC-DSB/02–20) and conformed to standards for the use of human subjects in research as outlined in the current Declaration of Helsinki. The study was registered at clinicaltrials.gov with the number NCT04503005.

**Table 1:** Participants’ baseline characteristics

	TRE 16:8 (*n* = 10)	TRE 14:10 (*n* = 10)	TRE 12:12 (*n* = 10)	ND (*n* = 10)
**Age (yr.)**	41.50 ± 13.19	44.40 ± 15.30	38.10 ± 13.34	41.70 ± 13.80
**Gender (f/m)**	5/5	6/4	5/5	4/6
**Body mass (kg)**	79.67 ± 19.33	72.17 ± 12.70	72.17 ± 12.00	68.34 ± 11.28
**BMI (kg/m** ^**2**^ **)**	26.78 ± 5.17	24.95 ± 3.00	24.67 ± 2.59	24.54 ± 3.82
**Fat mass (%)**	33.95 ± 7.22	37.39 ± 6.37	32.12 ± 5.41	31.25 ± 12.34

Data are presented as mean ± SD. BMI: body mass index; f: females; m: males. No baseline significant differences among groups

### Experimental design and diet protocols

**Fig. 2:**
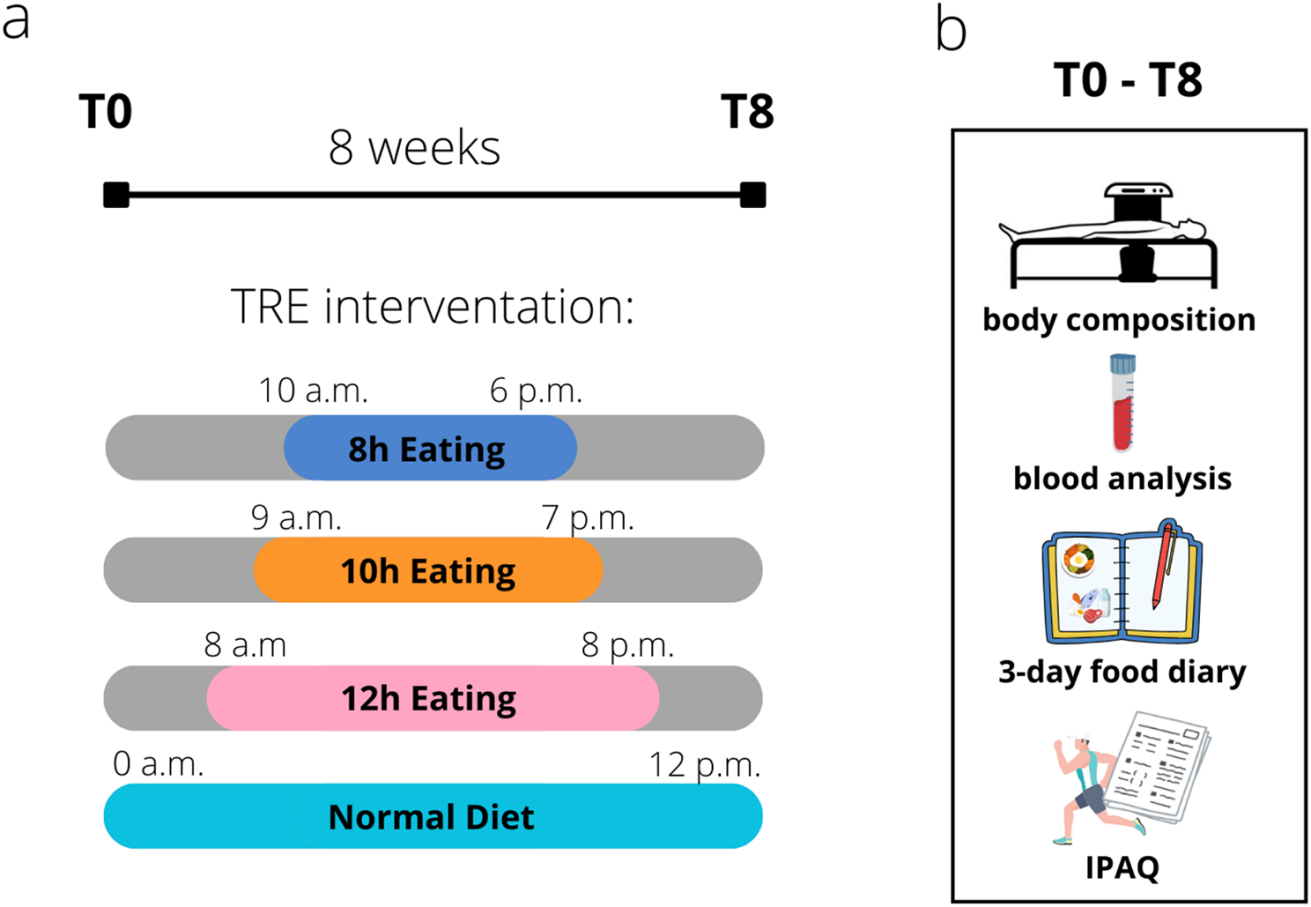
Experimental design. (**a**) The intervention lasted 8 weeks. The participants were randomized and assigned to either diet group after visit 1. (**b**) Measurements included body composition analysis via DEXA, blood analysis, completion of a 3-day food diary, and physical activity habits questionnaire. IPAQ: International Physical Activity Questionnaire

The study was designed as a randomized, controlled, parallel-arm trial. Participants were tested one week before the start (T0) and immediately after (T8) of the 8-week intervention (Fig. 2).

At the first visit (T0), participants were randomized into one of four groups: (1) TRE 16:8 [16 h of fasting and 8 h of feeding from 10 a.m. to 6 p.m.]; (2) TRE 14:10 [14 h of fasting and 10 h of feeding from 9 a.m. to 7 p.m.]; (3) TRE 12:12 [12 h of fasting and 12 h of feeding from 8 a.m. to 8 p.m.]; and (4) ND [no dietary restriction]. Participants were asked to maintain their habitual caloric intake, with the only restraint to limit food and caloric beverages in the assigned feeding window. The first day of the diet began 5–7 days after the first visit, following the analysis of a 3-day food diary [36–38]. Based on the diary results, participants were given suggestions on how to organize their meals to keep the distribution of calories and macronutrients as similar as possible to their usual habits. Participants also received weekly phone calls from an operator (AS) to optimize compliance and provide more recommendations if necessary.

### Measurements

All tests were performed between 07.00 a.m. and 10.00 a.m. during the overnight fasting. Participants were also asked to abstain from vigorous physical activity for 24 h prior to the test and to empty their bladders before each testing session to avoid any variation in body mass.

Body mass and height were measured to the nearest 0.1 kg and 1 cm, respectively, using a portable stadiometer (Wunder, Holtain Ltd., Crymych, UK). Body composition was subsequently assessed by dual-energy X-ray absorptiometry (DEXA, QDR 4500 W, Hologic Inc., Arlington, MA, USA). A certified team member (AP) performed the scans, with each participant lying supine and as steady as possible for approximately 7 min. Participants wore light clothing and removed any metal items that could interfere with the X-ray, including jewelry. A different operator (AS) analyzed the acquired images using the Hologic APEX Software (5.6.0.5). Total body fat mass and LST (i.e., free-fat mass without including bones) were assessed in kilograms. Visceral adipose tissue (VAT) was automatically estimated according to the manufacturer’s guidelines and following a standardized procedure used in previous studies [39, 40]. Specifically, the software identified VAT by measuring the total fat mass within a 5 cm-high region across the abdomen, situated approximately at the 4th and 5th vertebrae with the lower limit above the external ends of the iliac crests. Within this region, the software subtracts the subcutaneous fat from the total fat mass to generate VAT. The automatically generated lines used were manually adjusted if found inaccurate by the operator. Calibration of the DEXA was performed daily following the manufacturer’s procedures using a standard calibration block provided by the company (Hologic DXA Quality Control Phantom Lumbar Spine. Coefficient of Variation: 0.309%).

Approximately 12 mL of blood was collected from the antecubital vein for analysis of hemogram, lipid profile (total cholesterol, LDL, HDL, and triglycerides), fasting glucose, insulin, leptin, and testosterone. The samples were then processed and centrifuged, and the resultant serum was stored at − 80 °C until analysis. Insulin resistance was quantified using the homeostatic model assessment of insulin resistance (HOMA-IR). The HOMA-IR was computed by multiplying the fasting insulin and fasting glucose values, and then dividing the product by 405 [41]. A HOMA-IR value of less than 1 indicates high insulin sensitivity; values between 1 and 2.9 indicate normal insulin sensitivity; values greater than 2.9 suggest low insulin sensitivity and higher insulin resistance [41].

Participants filled in the validated Italian version of the International Physical Activity Questionnaire (IPAQ-SF [42]) to quantify weekly physical activity and hours spent sitting. IPAQ categorizes activity levels into three categories according to the weekly metabolic equivalent (MET): active (MET > 2520 MET), moderately active (700 < MET < 2519), and sedentary (< 700 MET) (Table 2).

Participants were asked to complete a 3-day food diary at baseline and after 8 weeks, reporting the type, quantity, brand, and timing of each meal [36–38]. Specifically, the pre-intervention diary included information from 3 days during the week preceding the start of the study, whereas the post-intervention diary covered 3 days immediately before the end of the 8th week. The average daily macronutrient intake was estimated using the free online software MyFitnessPal (myfitnesspal.com/it), which has previously demonstrated good relative validity, particularly for energy intake estimation [43].

**Table 2:** Participants’ habits of physical activity

	TRE 16:8	TRE 14:10	TRE 12:12	ND
Active	3	2	0	2
Moderately active	5	6	7	8
Sedentary	2	2	3	0

Number of subjects in each group

### Statistical analysis

To determine the number of participants to recruit, we conducted an a priori power analysis using the software G*Power (v.3.1.9.2). The target sample size was obtained based on our previous research [24] using fat mass as the primary outcome. To reach a power of 80% and an alpha risk of 5%, it was necessary to recruit a total of at least 36 participants to divide across the four groups. To account for potential dropouts during the study, we recruited a total of 40 participants.

The Shapiro–Wilk’s W test was used to assess normal distribution data and the one-way ANOVA test was used to ensure no baseline differences between groups. A two-way repeated-measures ANOVA (using time as the within-subject factor and diet type as the between-subject factor) was employed to assess differences between groups. Partial eta-squared (η_p_^2^) was calculated to measure the amount of variance of a dependent variable attributable to a given independent variable, considering the influence of the other independent variables present in the model. When a significant main or interaction effect was produced, the Bonferroni post-hoc correction was performed. Data analysis was performed using JASP software (JASP Team, 2024; version 0.18.3). All differences were considered significant at *p* < 0.05. Data are presented as mean ± standard deviation.

Given the possibility of drop-out and to verify the effects of diet compliance on outcomes, we performed both an intention-to-treat (ITT) analysis, including data from all participants, and a per-protocol (PP) analysis, including the participants who correctly completed the diet regimen.

## Results

The one-way ANOVA did not reveal any baseline differences among groups for any of the variables assessed.

Despite the participants’ weekly calls to monitor adherence to protocols, the analysis of the 3-day food diaries revealed that 8 participants did not fully adhere to the prescribed eating window. However, since we cannot confirm whether this eating pattern persisted throughout the 8 weeks, we first present the results based on the assigned intervention at the time of randomization (i.e., ITT), followed by the PP analysis, which includes only the participants who correctly completed the assigned treatment.

### Body composition

**Fig. 3:**
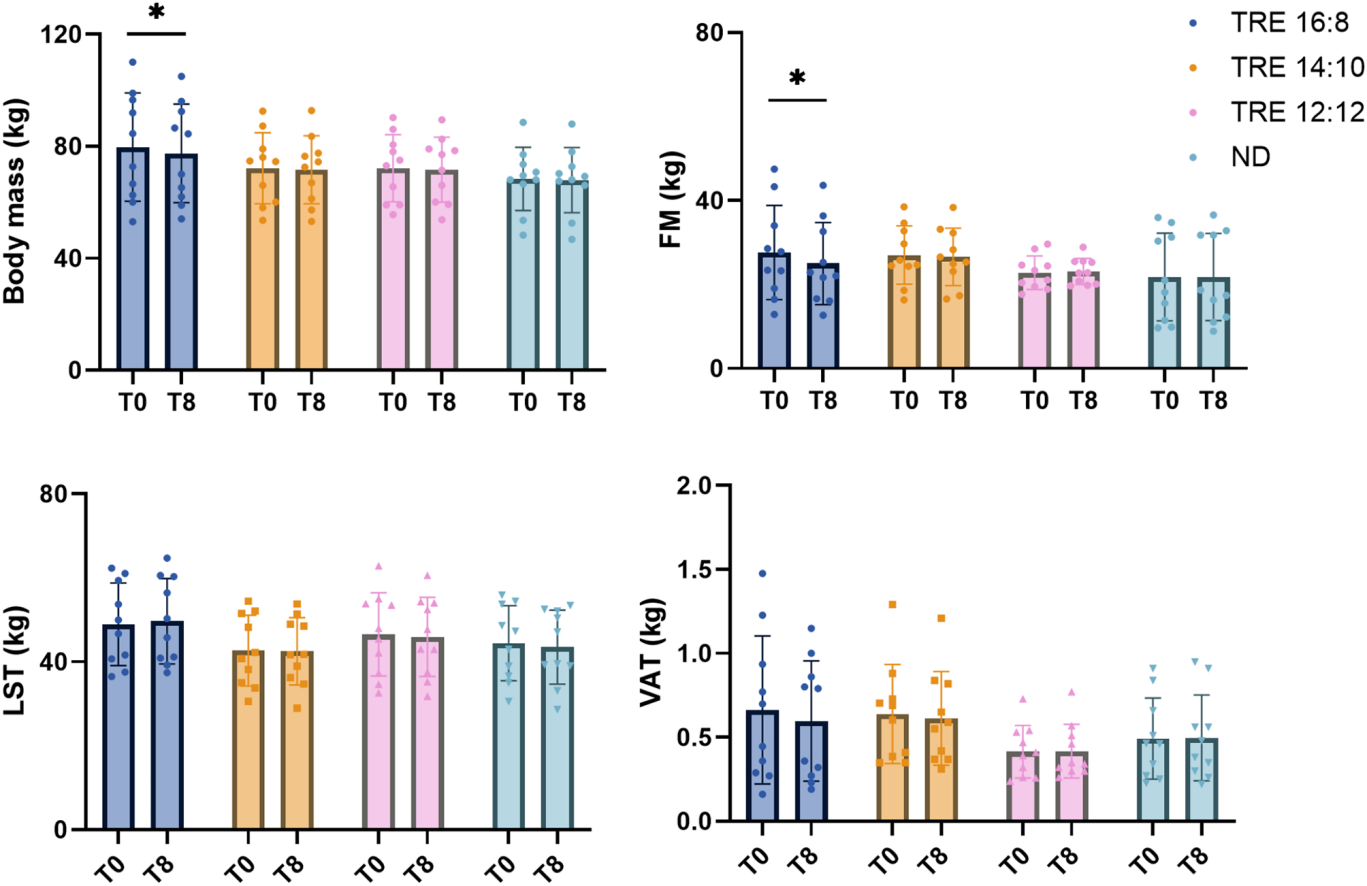
Body composition results. FM: fat mass (kg); LST: lean soft tissue (kg); VAT: visceral adipose tissue (kg); *: significant interaction time x group (*p* < 0.05)

Changes in body composition features are displayed in Fig. 3. After 8 weeks of intervention, the two-way ANOVA revealed a significant time × group interaction effect for body mass (*p* = 0.049, η_p_^2^ = 0.19) and absolute body fat mass (*p* = 0.003, η_p_^2^ = 0.32). Particularly, the body mass decreased by 2.46% ± 2.34% (from 79.67 to 77.42 kg) in TRE 16:8 (*p* = 0.003), whereas absolute body fat mass decreased by 8.65% ± 5.87% (from 27.64 to 25.01 kg) in TRE 16:8 (*p* = 0.001). In the same group, LST was maintained during the intervention (from 48.94 kg to 49.67 kg) and VAT did not significantly change after 8 weeks. For all the other groups, the two-way ANOVA did not reveal any significant changes in any of the parameters. The absolute and relative changes in all the variables studied within all groups are shown in the supplementary Table 1.

### Blood analysis

Data from blood biochemistry analysis are displayed in Table 3. No statistically significant time × group interaction and main effect were found for any of the blood markers analyzed. The absolute and relative changes in all the variables studied within all groups are shown in the supplementary Table 1.

**Table 3:** Major results of blood biochemistry analysis

	Groups	T0	T8	*P*-value group x time
CHOL tot (mg/dL)	TRE 16:8	187.70 ± 44.46	197.90 ± 57.18	0.815
	TRE 14:10	205.90 ± 31.97	202.10 ± 39.74	
	TRE 12:12	179.00 ± 26.50	175.20 ± 22.83	
	ND	197.70 ± 42.12	195.00 ± 32.86	
LDL (mg/dL)	TRE 16:8	122.60 ± 40.04	131.80 ± 44.56	0.376
	TRE 14:10	137.70 ± 23.33	135.60 ± 27.87	
	TRE 12:12	110.10 ± 21.04	105.20 ± 21.61	
	ND	125.90 ± 36.53	124.80 ± 29.10	
HDL (mg/dL)	TRE 16:8	63.30 ± 22.88	63.70 ± 26.00	0.084
	TRE 14:10	63.30 ± 14.68	57.00 ± 12.65	
	TRE 12:12	66.60 ± 15.69	65.20 ± 13.95	
	ND	68.70 ± 18.48	67.60 ± 15.45	
Triglyceride (mg/dL)	TRE 16:8	65.30 ± 26.84	63.40 ± 18.53	0.789
	TRE 14:10	90.30 ± 43.82	100.70 ± 40.52	
	TRE 12:12	66.30 ± 16.41	79.40 ± 30.23	
	ND	71.50 ± 20.77	81.20 ± 19.41	
Glucose (mg/dL)	TRE 16:8	91.90 ± 11.21	91.70 ± 11.80	0.303
	TRE 14:10	91.40 ± 6.40	88.60 ± 5.52	
	TRE 12:12	83.60 ± 5.58	82.70 ± 10.70	
	ND	91.80 ± 8.15	85.60 ± 10.02	
Insulin (mg/dL)	TRE 16:8	8.33 ± 6.12	9.08 ± 5.81	0.169
	TRE 14:10	8.55 ± 5.11	7.57 ± 3.75	
	TRE 12:12	6.22 ± 3.60	8.02 ± 2.79	
	ND	6.89 ± 2.85	5.99 ± 2.68	
HOMA-IR	TRE 16:8	1.85 ± 1.29	1.84 ± 1.19	0.203
	TRE 14:10	1.94 ± 1.17	1.52 ± 0.82	
	TRE 12:12	1.28 ± 0.73	1.43 ± 0.38	
	ND	1.60 ± 0.83	1.19 ± 0.72	
Leptin (mg/dL)	TRE 16:8	7.10 ± 9.19	5.87 ± 6.43	0.131
	TRE 14:10	9.80 ± 6.08	7.81 ± 5.43	
	TRE 12:12	5.04 ± 4.16	6.15 ± 4.05	
	ND	10.36 ± 12.35	9.67 ± 11.12	
Leptin/body mass (mg/dL·kg⁻¹)	TRE 16:8	0.09 ± 0.10	0.07 ± 0.07	0.113
	TRE 14:10	0.14 ± 0.09	0.11 ± 0.08	
	TRE 12:12	0.08 ± 0.08	0.10 ± 0.08	
	ND	0.15 ± 0.17	0.14 ± 0.15	
TST Free (mg/dL)	TRE 16:8	36.51 ± 34.45	33.02 ± 28.23	0.492
	TRE 14:10	29.61 ± 32.47	32.45 ± 31.13	
	TRE 12:12	44.60 ± 40.92	55.84 ± 67.64	
	ND	41.65 ± 35.09	42.90 ± 41.69	

Data are presented as mean ± SD

### Dietary intake

No significant differences were observed among all groups in total and per macronutrient energy intake (Table 4). Additionally, macronutrient intake was further analyzed in relation to body mass (grams per kilogram), including carbohydrate, fat, and protein intake. The statistical analysis did not reveal any time effect or interaction between group and time for any macronutrient intake per kilogram of body mass. The absolute and relative changes in all the variables studied within all groups are shown in the supplementary Table 1.

**Table 4:** Diet composition and macronutrient distribution

	Groups	T0	T8	*P*-value group x time
Total Energy (Kcal/day)	TRE 16:8	1565.85 ± 226.84	1258.96 ± 256.26	0.167
	TRE 14:10	1333.63 ± 274.67	1289.73 ± 504.95	
	TRE 12:12	1648.87 ± 484.15	1728.24 ± 584.98	
	ND	1467.67 ± 474.41	1409.04 ± 494.72	
Carbohydrates (kcal/day)	TRE 16:8	665.75 ± 246.37	541.81 ± 178.56	0.086
	TRE 14:10	642.33 ± 194.46	543.66 ± 283.09	
	TRE 12:12	685.88 ± 202.01	832.39 ± 414.67	
	ND	716.16 ± 224.28	623.42 ± 298.56	
Fat (Kcal/day)	TRE 16:8	553.00 ± 207.33	401.99 ± 141.04	0.491
	TRE 14:10	454.79 ± 208.02	430.36 ± 150.31	
	TRE 12:12	576.01 ± 237.78	662.39 ± 252.47	
	ND	509.99 ± 198.83	475.88 ± 153.82	
Protein (Kcal/day)	TRE 16:8	277.33 ± 64.91	238.22 ± 72.15	0.370
	TRE 14:10	278.80 ± 109.62	238.80 ± 102.61	
	TRE 12:12	300.67 ± 113.01	324.27 ± 80.85	
	ND	237.48 ± 68.41	239.85 ± 65.66	
**Macronutrient distribution normalized for body mass**				
Carbohydrates (g/day·kg)	TRE 16:8	2.18 ± 0.55	1.86 ± 0.66	0.115
	TRE 14:10	2.39 ± 0.99	2.05 ± 1.42	
	TRE 12:12	2.45 ± 0.61	3.04 ± 1.40	
	ND	2.79 ± 1.14	2.55 ± 1.60	
Fat (g/day·kg)	TRE 16:8	0.85 ± 0.51	0.78 ± 0.46	0.537
	TRE 14:10	0.73 ± 0.41	0.63 ± 0.22	
	TRE 12:12	0.90 ± 0.35	1.04 ± 0.41	
	ND	0.88 ± 0.47	0.93 ± 0.53	
Protein (g/day·kg)	TRE 16:8	0.92 ± 0.27	0.83 ± 0.36	0.342
	TRE 14:10	1.01 ± 0.49	0.83 ± 0.31	
	TRE 12:12	1.05 ± 0.38	1.31 ± 0.21	
	ND	0.90 ± 0.30	0.93 ± 0.35	

Data are presented as mean ± SD

### Results of per-protocol analysis

The data from the PP analysis included 8 participants in TRE 16:8, 5 in TRE 14:10, 9 in TRE 12:12, and 10 in ND. Significant interactions among groups for body composition were maintained despite the different number of participants in each group. Specifically, we observed a significant group × time interaction for body mass (*p* = 0.016, η_p_^2^ = 0.30) and absolute body fat mass (*p* = 0.004, η_p_^2^ = 0.37), while LST did not significantly change. Notably, in TRE 16:8, body mass decreased by 2.73% ± 2.57% (from 84.28 to 81.68 kg, *p* = 0.003) and the absolute fat mass decreased by 9.78% ± 6.04% (from 29.56 to 26.49 kg, *p* = 0.002).

None of the biochemical parameters were affected by the diet protocols, showing no main effects or interactions. Additionally, overall physical activity levels and daily caloric intake remained unchanged after the intervention.

## Discussion

Dietary habits extend beyond basic survival needs, encompassing significant social and cultural dimensions [44]. In this regard, recently, some authors have tried to re-evaluate and articulate the core characteristics of traditional dietary patterns globally through the “Planeterranean” approach, which integrates diet, culture, and health promotion [9]. While several factors are embedded in dietary habits and their effects on health, they are often not fully considered [17], including the duration and length of daily fasting period. Fasting plays a crucial role in many cultural and religious contexts, often seen as more than just an act of food deprivation. It serves as a practice of spiritual discipline, symbolizing purification, self-control, and a deepened connection with the divine. In many traditions, fasting is associated with spiritual renewal and moral introspection, making it a key element of religious observance. In Islam, for instance, fasting during the holy month of Ramadan is one of the Five Pillars of faith, encouraging not only physical restraint from food and drink but also from sinful behaviors. It is a time for self-purification, spiritual reflection, and empathy toward those who are less fortunate [45]. Similarly, in Christianity, fasting during Lent is associated with penance and self-examination, preparing the faithful for Easter by fostering humility and spiritual growth [46, 47]. Jewish traditions, such as fasting during Yom Kippur, focus on atonement and repentance, while in Hinduism, fasting is often performed to purify the mind and body, fostering a sense of mental clarity and spiritual elevation [48]. In Buddhism, fasting is linked to practices of mindfulness and self-control, forming part of broader meditative and spiritual exercises [49].

Cultural fasting is not limited to religious observance. In many agrarian societies, involuntary fasting has historically been tied to the rhythms of agricultural life. Periods of food scarcity, particularly during winter or droughts, led to cycles of feasting and fasting that became embedded in cultural rituals. For instance, in pre-industrial European societies, the scarcity of food during late winter or early spring often led to fasting traditions that coincided with religious observances like Lent [50]. This seasonal fasting was not only a survival strategy but also a way of sanctifying hardship, linking it to religious and cultural meaning. Similarly, in parts of Africa and Asia, fasting practices developed as adaptive responses to environmental conditions, later intertwining with spiritual beliefs [51]. These involuntary periods of fasting became ritualized over time, highlighting the cultural significance of food cycles.

Thus, fasting serves as both a voluntary and involuntary practice shaped by religious and environmental factors, emphasizing its profound impact on cultural identity, community solidarity, and spiritual life. In addition to all that, fasting periods, embedded in almost all religious precepts, may explain part of the positive role of the Mediterranean diet on health. For example, it has been highlighted that the many days of fasting prescribed by the Orthodox Christian dietary rules represent an important component of the benefits given by the general traditional healthy (and sustainable) Mediterranean diet of Crete [52]. Therefore, our study aimed to study the effects of 8 weeks of different lengths of fasting on body composition and health-related biomarkers in a group of healthy and non-trained individuals. To the best of our knowledge, this is the first study to compare different fasting durations in a healthy and non-trained cohort, as most research has focused on individuals with obesity or metabolic impairments [26, 27, 33, 53, 54].

Our results suggest that a 16-hour fasting period reduces body mass and body fat mass while maintaining lean body mass. It is crucial to emphasize that participants were instructed to maintain stable caloric intake during the intervention, which is consistent with our previous studies on TRE [24, 55]. A slight reduction in energy intake was expected, as moving meals closer together may suppress appetite, increase stomach fullness, and reduce the desire to eat [56]. However, the reduction in caloric intake was not statistically significant, suggesting that the different time distribution of meals (i.e., fasting hours) might explain per se body composition improvement observed in TRE 16:8. Indeed, upon the first 12–14 h of fasting liver glycogen stores are depleted [29, 57]. As a result, fatty acids are mobilized from adipocytes into the bloodstream and then into the hepatocytes, where they can be converted into ketone bodies (KB) as a new energy source. Although we did not measure KBs’ blood concentration in this study, others have observed that KBs are already produced after 14 h of fasting and increase with the duration of fasting [58]. As proposed by Mattson and colleagues [19], during IF protocols, glucose levels rise during and for several hours after food intake, then decline and remain low until the next day’s meal. Meanwhile, KBs levels increase during the final 6–8 h of the 18-hour fasting period [19]. In the 16:8 group, we observed a (non-significant) reduction in carbohydrate consumption, which might have led to a reduced glucose level and might have favoured the production of KB during the longer fasting window. Considering the anorexigenic effects of KB [59], we can hypothesize that if this condition is repeated over time, it may lead to a significant reduction of total energy daily intake [60] and, thus, to a reduction in body fat mass.

The TRE 14:10 group did not experience changes in body composition. These results contrast with previous research, which reported reductions in body mass and fat mass following a TRE 14:10 regimen [33, 53]. However, these studies involved participants with overweight and obesity and reported greater caloric restriction compared to our research. Our analysis of the food diaries revealed that participants in the TRE 14:10 group had more difficulty following the prescribed eating pattern. Specifically, participants were inconsistent in eating at the same time (i.e., they changed the length of fasting), and two participants spontaneously moved to a 16-hour fasting scheme in the final weeks of the trial. Therefore, we hypothesize that the differences between our results and previous findings may be due to both the baseline conditions of subjects (i.e., not overweight vs. overweight) and our participants’ inconsistent adherence to the fasting schedule.

Another interesting finding is the absence of changes in VAT. Although scientific findings are controversial, previous studies on TRE have found that a portion of body fat loss occurs in visceral fat [53, 61]. We hypothesize that this discrepancy may be due to our participants’ baseline fat mass levels compared to those in previous studies and because our intervention was 4 weeks shorter in duration. Individuals with greater VAT typically lose more visceral fat in relation to the total fat mass when undergoing weight control interventions [62]. Our participants ranged from 0.16 to 1.48 kg of VAT while most of the studies that observed a significant reduction in VAT presented with more than 3 kg at baseline [62]. Moreover, most of the studies applied 12 weeks of intervention to observe a significant reduction in VAT, which might suggest that 8 weeks of TRE are not sufficient to induce such a result.

Our results showed that TRE did not affect any of the blood parameters measured across all groups. It is plausible that the extent of body mass loss observed in our study was not substantial enough to impact these outcomes. Evidence suggests that improvements in plasma lipid concentrations and glucoregulatory factors generally require more than 5% body mass loss [6, 63]. However, it has been shown that the metabolic effects may occur even without body mass loss [64]. Thus, further research is needed to assess the impact of body mass loss in a TRE protocol on cardiometabolic outcomes. Additionally, the absence of improvement in blood parameters may result from different inclusion criteria of participants, as our study included metabolically healthy humans, whereas previous studies often involved individuals with obesity or metabolic alterations or have included exercise in the intervention [24, 33, 53]. Our results are in accordance with other studies reporting no improvement in lipid profile, fasting glucose, and fasting insulin [26, 27, 54, 61, 65]. We also hypothesized that the chosen eating window in this study (from 10 a.m. to 6 p.m.) may not be optimal for producing evident metabolic effects in healthy individuals. Although the prescribed eating window is likely more attractive and more amenable to long-term adherence, a recent study [65] showed that shifting the eating window to earlier in the day (from 6 a.m. to 3 p.m.) resulted in improved fasting glucose and insulin sensitivity, reduced inflammation, and increased gut microbial diversity compared to participants who followed TRE between 11 a.m. and 8 p.m. Additionally, a recent systematic review on the metabolic effects of TRE indicated that this dietary regime reduced fasting insulin and HOMA-IR while fasting glucose is decreased only in early TRE among individuals with obesity [25]. This difference in metabolic outcomes may be explained by the alignment of TRE with the body’s circadian rhythms. Insulin secretion and sensitivity are also under circadian regulation: these parameters are increased early in the day and drop in the evening [66]. Studies have shown that insulin sensitivity and beta-cell responsiveness are higher in the morning, leading to better glucose control when food intake is concentrated in this window of time [67]. Thus, the reduction in insulin sensitivity in the evening may explain the different outcomes in glucose concentrations when TRE is performed early or later in the day.

Another factor that should be considered is the presence or not of physical activity. In our previous studies, we found evident improvements in triglycerides, insulin, and glycemia in strength athletes [24, 60] and partially in cyclists [55] who underwent the same dietary protocol of TRE and were free of metabolic diseases. Recent research also found that inactive women with obesity who performed high-intensity training combined with early TRE showed greater improvement in total cholesterol, triglyceride, insulin, HOMA-IR, and glucose compared to a control group that only followed TRE [68]. These findings suggest that the physical exercise component is crucial for enhancing the effects of TRE on these metabolic parameters. Indeed, some researchers have already put forward this hypothesis by suggesting that exercise and fasting may have mutually reinforcing effects [66]. Regular exercise enhances insulin sensitivity, which can lower fasting glucose levels, and improves free fatty acid concentrations and lipid profile [69]. These are precisely the metabolic markers that did not show significant changes in our study, potentially due to the absence of a physical exercise component. However, further research is needed to explore the interaction between exercise and TRE.

### Strengths and limitation

A few strengths and limitations should be mentioned in relation to this study. Strengths of the study included the use of DXA, which is acknowledged as the gold standard in research due to its accurate and reliable predictions of fat mass and LST [70]. This allows for a more precise evaluation of body composition changes following the intervention. Moreover, to ensure the robustness of our findings, we used both ITT and PP analyses. This approach is recommended for randomized trials as it preserves the statistical power of the original sample [71]. This dual approach allowed us to compare the overall effects of the interventions as well as the specific effects on compliant participants. Both ITT and PP analyses showed consistent results, suggesting that TRE 16:8 is effective in reducing body mass primarily through a decrease in fat mass, while fewer hours of fasting seem not sufficient to produce significant results.

Nevertheless, some limitations should be considered. First, adherence to TRE was evaluated by using a 3-day diary of food, which monitored only the last 3 days of the intervention. Despite the weekly calls to remind and encourage participants to adhere to the protocol, it is possible that participants overstated their compliance. Likewise, self-reported nutrient intake may be inaccurate, potentially resulting in either overestimation or underestimation of the caloric intake. Second, to improve compliance with TRE, we allowed low-energy drinks, including coffee, during fasting hours. Although these drinks have no caloric impact, the caffeine content may affect circadian rhythms [72], which is one of the principles of TRE. Specifically, caffeine can delay the phase of the human circadian clock by altering the melatonin rhythm [72]. Since melatonin is a hormone that regulates sleep-wake cycles and influences various metabolic processes [73], disruption in its secretion may negatively affect energy balance and fat storage, potentially impacting body composition. Third, the duration of the intervention (i.e., 8 weeks) may be insufficient to reveal any significant effects of TRE on metabolic biomarkers or body composition, particularly with the 14-hour fasting protocol. Thus, additional research should consider extending the duration of the intervention to provide information about the long-term impacts of TRE. In addition, our study included individuals with a broader age range (18–65 year.); however, age-related physiological differences may influence body composition and metabolic responses, highlighting the necessity for future studies to investigate the potential differential effects of TRE across younger and older populations.

Lastly, the relatively small number of participants included in the PP analysis, especially in the TRE 14:10 group, may limit the conclusiveness of our findings.

## Conclusions

Since agricultural revolution (10,000 years ago) and for thousands of years when artificial light was challenging to obtain or expensive, the circadian inner clock, together with the natural light/dark cycle and its effects on the central nervous system, regulated the daily rhythms in physiology and behaviours, including the activity/rest and feeding/cycle. For our ancestors, even after agricultural revolution, food was scarce and mainly consumed during daylight hours: these social and environmental conditions led to long hours of overnight fasting. For these reasons, fasting length in humans should be considered when eating behaviours are analysed.

Based on our findings, clinicians should consider recommending an 8-hour daily window (16 h of fasting) for improving body mass in healthy individuals, as this dietary approach decreases fat mass while maintaining LST. Notably, our results suggest that fasting 16 h does not significantly reduce caloric intake, letting us suppose that the observed improvements are more likely associated with meal timing rather than a reduction in calorie consumption. However, it should be noted that TRE, without any other interventions such as physical exercise, does not improve lipid profiles in metabolically healthy individuals. Thus, in a Planeterranean view, to exert its positive effects on healthy individuals, fasting must be associated with physical activity and must be at least 16 h in length.

## Electronic supplementary material

Below is the link to the electronic supplementary material.

Supplementary Material 1

## Data Availability

The datasets used and/or analysed during the current study are available from the corresponding author on reasonable request.

## Abbreviations

CCC: Continuous calorie restriction
DEXA: Dual-energy X-ray absorptiometry
IF: Intermittent fasting
IPAQ: International Physical Activity questionnaire
ITT: Intention-to-treat analysis
KB: Ketone bodies
MET: Metabolic equivalent
ND: Normal diet group
PP: Per-protocol
TRE: Time-restricted eating
VAT: Visceral adipose tissue
WHO: World Health Organization
