# Supplementary material for: Impact of daily fasting duration on body composition and cardiometabolic risk factors during a time-restricted eating protocol: a randomized controlled trial
Source: J Transl Med. 2024 Nov 29;22:1086. doi: 10.1186/s12967-024-05849-6 (PMC11607941; doi:10.1186/s12967-024-05849-6)
Supplement: Supplementary file 1 — Supplementary Material 1 [file 12967_2024_5849_MOESM1_ESM.docx]

Supplementary Table 1. Relative and absolute changes in body composition, blood analysis and diet composition.

|  | TRE 16:8 | TRE 14:10 | TRE 12:12 | ND |
| --- | --- | --- | --- | --- |
| **BODY COMPOSITION** | | | | |
| **Body mass** | -2.46 ± 2.34 (%)  -2.25 ± 2.18 (kg) | -0.7 ± 2.18 (%)  -0.59 ± 1.69 (kg) | -0.6 ± 2.18 (%)  -0.49 ± 1.61 (kg) | -0.74 ± 1.14 (%)  -0.43 ± 0.68 (kg) |
| **Fat mass** | -8.65 ± 5.87 (%)  -2.63 ± 2.21 (kg) | -1.42 ± 3.65 (%)  -0.40 ± 0.91 (kg) | 2.20 ± 7.90 (%)  0.31 ± 1.81 (kg) | 0.56 ± 9.50 (%)  -0.05 ± 1.87 (kg) |
| **LST** | 1.47 ± 2.56 (%)  0.73 ± 1.30 (kg) | -0.22 ± 4.02 (%)  -0.18 ± 1.65 (kg) | -0.98 ± 4.47 (%)  -0.56 ± 1.70 (kg) | -2.02 ± 5.07 (%)  -0.91 ± 2.14 (kg) |
| **VAT** | -6.16 ± 16.22 (%)  -0.07 ± 0.13 (kg) | -3.24 ± 12.87 (%)  -0.03 ± 0.07 (kg) | 2.00 ± 12.33 (%)  0.01 ± 0.05 (kg) | 1.20 ± 13.97 (%)  0.01 ± 0.06 (kg) |
| **BLOOD ANALYSIS** | | | | |
| **CHOL tot** | 5.15 ± 14.11 (%)  10.2 ± 29.15 (mg/dL) | -2.08 ± 9.40 (%)  -3.80 ± 19.14 (mg/dL) | -1.57 ± 8.27 (%)  -3.80 ± 15.89 (mg/dL) | -0.20 ± 8.28 (%)  -2.70 ± 15.50 (mg/dL) |
| **LDL** | 9.27 ± 21.00 (%)  9.2 ± 28.19 (mg/dL) | -1.53 ± 12.10 (%)  -2.10 ± 16.57 (mg/dL) | -4.13 ± 11.63 (%)  -4.9 ± 13.02 (mg/dL) | -0.65 ± 6.60 (%)  -1.10 ± 4.70 (mg/dL) |
| **HDL** | -0.52 ± 9.92 (%)  0.4 ± 6.51 (mg/dL) | -9.28 ± 8.15 (%)  -6.3 ± 5.72 (mg/dL) | -1.28 ± 9.09 (%)  -1.40 ± 6.62 (mg/dL) | -0.65 ± 6.60 (%)  -1.10 ± 4.70 (mg/dL) |
| **Triglyceride** | 7.27 ± 35.53 (%)  -1.90 ± 18.41 (mg/dL) | 27.54 ± 62.37 (%)  10.40 ± 54.58 (mg/dL) | 25.61 ± 63.76 (%)  13.10 ± 33.79 (mg/dL) | 22.47 ± 49.70 (%)  9.70 ± 24.11 (mg/dL) |
| **Glucose** | -0.05 ± 8.18 (%)  -0.20 ± 7.33 (mg/dL) | -2.88 ± 5.53 (%)  -2.80 ± 4.96 (mg/dL) | -1.08 ± 10.91 (%)  -0.90 ± 9.17 (mg/dL) | -6.58 ± 8.65 (%)  -6.20 ± 8.16 (mg/dL) |
| **Insulin** | 26.25 ± 64.60 (%)  0.75 ± 2.53 (mg/dL) | 18.26 ± 76.52 (%)  -0.98 ± 4.60 (mg/dL) | 71.40 ± 140.11 (%)  1.80 ± 3.39 (mg/dL) | -12.39 ± 20.36 (%)  -0.90 ± 1.38 (mg/dL) |
| **HOMA-IR** | 13.02 ± 56.27 (%)  -0.01 ± 0.52 | 3.91 ± 66.01 (%)  -0.41 ± 1.02 | 59.15 ± 148.84 (%)  0.15 ± 0.81 | -25.92 ± 20.99 (%)  -0.42 ± 0.34 |
| **Leptin** | -2.80 ± 27.50 (%)  -1.23 ± 3.33 (mg/dL) | -20.35 ± 28.46 (%)  -1.99 ± 3.46 (mg/dL) | 39.54 ± 56.64 (%)  1.11 ± 2.32 (mg/dL) | 19.03 ± 56.11 (%)  -0.69 ± 2.51 (mg/dL) |
| **Leptin/body mass** | -0.28 ± 28.57 (%)  -0.01 ± 0.04 | -20.19 ± 26.98 (%)  -0.03 ± 0.05 | 40.38 ± 56.99 (%)  0.02 ± 0.03 (mg/dL ·kg⁻¹)) | 19.88 ± 56.24 (%)  -0.01 ± 0.03 (mg/dL·kg⁻¹) |
| **TST Free** | 9.74 ± 39.54 (%)  -3.49 ± 8.38 (mg/dL) | 76.72 ± 252.49 (%)  2.84 ± 14.07 (mg/dL) | 17.23 ± 44.55 (%)  11.24 ± 37.17 (mg/dL) | -0.31 ± 30.02 (%)  1.25 ± 13.84 (mg/dL) |
| **DIET COMPOSITION** | | | | |
| **Total Energy** | -18.23 ± 20.27 (%)  -276.20 ± 321.57 (kcal/day) | -1.59 ± 29.99 (%)  -11.03 ± 404.88 (kcal/day) | 6.77 ± 23.82 (%)  79.37 ± 368.66 (kcal/day) | -3.11 ± 20.30 (%)  -52.77 ± 307.46 (kcal/day) |
| **Carbohydrates** | -11.85 ± 33.30 (%)  -123.94 ± 236.52 (kcal/day) | -15.73 ± 27.97 (%)  -98.67 ± 204.92 (kcal/day) | 19.09 ± 42.69 (%)  146.51 ± 287.89 (kcal/day) | -9.64 ± 37.32 (%)  -92.74 ± 287.24 (kcal/day) |
| **Fat** | -3.99 ± 38.92 (%)  -60.33 ± 242.96 (kcal/day) | -3.30 ± 35.73 (%)  -52.79 ± 233.34 (kcal/day) | 29.55 ± 68.20 (%)  86.38 ± 305.75 (kcal/day) | 3.16 ± 15.04 (%)  16.01 ± 68.76 (kcal/day) |
| **Protein** | -12.75 ± 25.68 (%)  -39.11 ± 66.17 (kcal/day) | -9.92 ± 32.11 (%)  -40.00 ± 103.39 (kcal/day) | 20.70 ± 52.83 (%)  23.60 ± 120.32 (kcal/day) | 5.56 ± 25.52 (%)  2.37 ± 72.20 (kcal/day) |

Data are presented as mean ± SD.
